# Chromosome diversity in Buthidae and Chactidae scorpions from Brazilian fauna: Diploid number and distribution of repetitive DNA sequences

**DOI:** 10.1590/1678-4685-GMB-2022-0083

**Published:** 2023-05-15

**Authors:** Juliana Figueiredo Lima, Leonardo Sousa Carvalho, Marcos André Carvalho, Marielle Cristina Schneider

**Affiliations:** 1 Universidade de São Paulo (USP), Instituto de Biociências, Departamento de Zoologia, Programa de Pós-Graduação em Zoologia, São Paulo, SP, Brazil.; 2 Universidade Federal do Piauí (UFPI), Floriano, PI, Brazil.; 3 Universidade Federal de Mato Grosso (UFMT), Departamento de Biologia e Zoologia, Cuiabá, MT, Brazil.

**Keywords:** Cot-DNA fraction, cytogenetic, heterochromatin, rDNA genes, telomere sequence

## Abstract

In this work, we analyzed cytogenetically eight Chactidae and Buthidae, including the localization of repetitive DNA sequences. The chactids possess monocentric chromosomes and the highest diploid numbers (2n=50 in *Brotheas amazonicus*, 2n=36 in *Chactopsis amazonica*, 2n=30 in *Neochactas* sp.) when compared with buthids (2n=10 in *Tityus bahiensis*, 2n=14 in *Tityus apiacas* and *Tityus metuendus*, 2n=18 in *Tityus aba*, 2n=26 in *Ischnotelson peruassu*). The localization of rDNA genes and (TTAGG)n sequences exhibited a conserved pattern of two terminal/subterminal ribosomal cistrons and terminal telomere signals. However, the comparison between the data of C-banding, DAPI after FISH and Cot-DNA fraction indicated a variable quantity and distribution of these regions, as follow: (i) positive heterochromatin and Cot-DNA signals (*B. amazonicus* and *I. peruassu*), (ii) small blocks of heterochromatin with large Cot-DNA signals (*T. metuendus*), (iii) positive heterochromatic regions and absence of Cot-DNA signals (*T. aba* and *T. apiacas*), and (iv) negative heterochromatin and Cot-DNA signals (*T. bahiensis*). Therefore, our results revealed that there still is not a clear relation between quantity of heterochromatin and presence of monocentric or holocentric chromosomes and occurrence of chromosomal rearrangements, indicating that repetitive regions in scorpions must be analyzed using different cytogenetic approaches.

## Introduction

The cytogenetic information on scorpions has a greatly improved in the last 20 years, from approximately 60 studied species to 270 (Schneider et al., 2023). However, these data are still limited to 10% of the 2749 taxonomically identified species, which are grouped into 11 of the 22 families recognized (Rein, 2023; Schneider *et al.*, 2023). In the Brazilian scorpion fauna, cytogenetic studies are also neglected, with chromosomal data for only 27 species. In contrast to this scenario, scorpions have many cytogenetic particularities, such as the occurrence of monocentric and holocentric chromosomes, high intraspecific and interspecific variability of diploid number, and meiosis with achiasmatic behavior of the chromosomes and presence of multivalent chromosomal chains (Mattos et al., 2018; Ubinski et al., 2018; Adilardi et al., 2020; Šťáhlavský et al., 2020, 2021; Schneider *et al.*, 2023). The knowledge of all these characteristics can help to hypothesize about the evolution of chromosomes with localized and diffuse-kinetochore, the relationship between the chromosome structure/organization and the putative chromosomal rearrangements, and the mechanism responsible for the genetic variability in scorpions.

Within the Brazilian scorpion fauna there are cytogenetic data for three families: Buthidae, Bothriuridae and Chactidae. The buthids are worldwide distributed (Stockmann and Ythier, 2010) and present most cytogenetic data, with 166 species already characterized. In this family the diploid numbers range from 2n=5 to 2n=56, including genera with conserved chromosome number, such as *Androctonus*, 2n=24 (11 species) and *Compsobuthus*, 2n=22 (seven species), or very variable, such as *Tityus*, 2n=5-32 (30 species) and *Uroplectes*, 2n=16-48 (10 species). The presence of holocentric chromosomes is exclusive of this family (Schneider et al., 2023). The Bothriuridae and Chactidae have distribution restricted to South America (Stockmann and Ythier, 2010). The bothriurids possess chromosomal records for 10 species included in three genera and exhibited a predominance of high diploid numbers, 2n=42-50, with only two exceptions (2n=28 and 2n=36). In Chactidae, there is only a brief description of diploid number for *Brotheas amazonicus*, with 2n=50 (Ferreira, 1968). Differing from Bothriuridae, for this last family the presence of monocentric chromosomes was still not confirmed. 

Repetitive DNA sequences constitute a large part of the genome of eukaryotes and are found mainly in regions of low or absent genetic recombination (e.g. centromeric, telomeric and heterochromatic regions) (Charlesworth et al., 1994; Kejnovsky et al., 2009; Cabral-de-Mello et al., 2010). Repetitive DNA is formed by equal or similar sequences that may be distributed in tandem or dispersed throughout the genome. In tandem repetitive sequences include microsatellites, minisatellites, satellite DNAs, and multigene families, such as ribosomal (rDNAs) and histone genes. The dispersed repetitions include DNA transposons and retrotransposons (Charlesworth *et al.*, 1994). Repeated sequences have been recognized as valuable for the chromosome characterization of species or populations, identification of chromosomal rearrangements and homologous chromosomes in holocentric species and mechanisms involved in the diversification of the genomes (Flavel, 1986; Martins et al., 2004; Mravinac et al., 2004; Pamoleque and Lorite, 2008; Ferreira et al., 2010; Roa and Guerra, 2012; Schneider et al., 2013; Pita et al., 2014).

Studies on repetitive DNA in scorpions have been focused on the location of the ribosomal genes (about 110 species), which are most often found in the interstitial or terminal region of two chromosomes, and the telomeric sequences (about 40 species) (Schneider and Cella, 2010; Adilardi et al., 2014, 2015, 2016, 2020; Mattos et al., 2014, 2018; Almeida et al., 2017; Šťáhlavský et al., 2018, 2020, 2021; Ubinski et al., 2018). In a comparative analysis about the quantity and distribution of constitutive heterochromatin in 11 species of buthid scorpions using C-banding and fluorochrome staining, Mattos *et al.* (2013) suggested that the species with the highest amount of constitutive heterochromatin had the lowest rates of chromosomal rearrangements.

Scorpions are particularly interesting for studies of repetitive DNAs, given the occurrence of different types of chromosomes (monocentric and holocentric) and highly divergent diploid numbers in closely-related species. The high rates of chromosomal rearrangements recorded in the scorpions, along with the achiasmatic mode of meiosis, may also contribute to evaluate the influence of repetitive sequences on the structure, function, and stability of the chromosomes. Considering all this, in this study, we analyzed cytogenetically Brazilian scorpions of the families Chactidae and Buthidae, including the localization of the heterochromatin and repetitive DNA sequences (multigene family and/or satellite DNA). Our study covered eight species belonging to five different genera. This work is descriptive, but it also provides comparisons via different techniques to identify the heterochromatin as well as among species with monocentric and holocentric chromosomes and with different types of chromosomal rearrangements.

## Material and Methods

We analyzed three chactid species - genera *Brotheas*, *Chactopsis* and *Neochactas*, and five buthids - genera *Ischnotelson* and *Tityus* scorpions (Table 1). The species studied were assigned to well-described species documented in reliable taxonomic literature (i.e. Lourenço, 2002a; Esposito et al., 2017). Nevertheless, the specimens were determined as *Tityus apiacas*, according to Lourenço (2002b) and collection locality, and considering the lack of distinctive characters of other described scorpions of subgenus *Atreus*. The vouchers were deposited in the Brazilian arachnological collections of the Centro de Coleções Taxonômicas of the Universidade Federal de Minas Gerais (CTUFMG, curator A.J. Santos), Belo Horizonte, state of Minas Gerais, Coleções Zoológicas of the Universidade Federal de Mato Grosso, (CZUFMT, curator A. Chagas-Jr.), Cuiabá, state of Mato Grosso and Coleção de História Natural of the Universidade Federal do Piauí (CHNUFPI, curator L.S. Carvalho), Floriano, state of Piauí.

**Table 1 -:** Chactidae and Buthidae scorpions analyzed in this study, including the numbers of specimens, their sampling localities in Brazilian states, the cytogenetic information with the percentages and number of cells (parentheses) analyzed. AM = Amazonas. BA = Bahia. CE = Ceará. MG = Minas Gerais. MT = Mato Grosso. SP = São Paulo. RO = Roraima. II = bivalent. C = chromosome chain. IV = chain of four chromosomes. VN = variable number. + = positive bands. ± = tenuous bands. - = negative bands. I = interstitial. ST = subterminal. T = terminal.

	Species	Number of individuals	Collection localities	Diploid number	Postpachytene configuration	C-Band and DAPI after FISH	28S rDNA	Telomere sequence	Species-specific Cot-DNA	*T. metuendus* Cot-DNA
Chactidae										
	*Brotheas amazonicus* Lourenço, 1988	4♀, 4♂ 2♂	Reserva Adolpho Ducke (2°57’S, 59°55’W), Manaus, AM Novo Airão (2°42’S, 60°56’W), Manaus, AM	**50**	**25II** 100 (49) **25II** 88 (75) **23II+IV** 12.0 (75)	**C-band: + T** 51.8 (87) **DAPI: + T and I** 85.4 (179)	**2 ST** 30.2 (43)	**T** 44.9 (49)	±T 12.6 (87)	±I 17.6 (51)
	*Chactopsis amazonica* Lourenço & Francke, 1986	1♂	Reserva Adolpho Ducke (2°57’S, 59°55’W), Manaus, AM	**36**	**18II** 86.6 (75) **16II+IV** 13.3 (75)	**C-band: -** 100 (25) **DAPI: -** 100 (138)	**2 T** 14.7 (95)	**T** 60.0 (35)	-	
	*Neochactas* sp.	3♀, 2♂	Estância Ecológica SESC Tepequém (3°45’N, 61°42’), Amajari, RO	**30**	**15II** 33.3 (18)	**C-band: -** 100 (22) **DAPI: -** 100 (11)			-	
Buthidae										
	*Ischnotelson peruassu* Esposito, Yamaguti, Souza, Pinto da Rocha & Prendini, 2017	1♀, 2♂	Itacarambi (16°30’S, 41°30’W), MG	**26**		**DAPI: +T** 88.4 (121)			**+T** 22.5 (31)	±I 15.7 (89)
	*Tityus aba* Candido, Lucas, de Souza, Diaz & Lira-da-Silva, 2005	2♀, 1♂	Distrito de Catalés (13°18’S, 41°51’W), Abaíra, BA	**18**	**9II** 100 (11)	**DAPI: +T and ±I** 100 (18)		**T** 47.5 (40)	-	-
	*Tityus apiacas* Lourenço, 2002	1♀, 5♂	Cláudia (11°34’S, 55°17’W), MT	**14**	**7II** 20 (155) **VNII+VNC** 80.0 (155)	**DAPI: +T and ±I** 91.0 (235)	**2 T** 18.0 (72)			-
	*Tiyus bahiensis* (Perty, 1833)	5♂	Estação Ecológica de Itirapina (22º13’S, 47º54’ W), SP	**10**	**5II** 88.5 (35)	**DAPI: -** 100 (29)				±I 21.4 (28)
	*Tityus metuendus* Pocock, 1897	1♀, 4♂ 1♀, 2♂	Universidade Federal do Amazonas (3°06’S, 59°58’W), Manaus, AM Reserva Adolpho Ducke (2°57’S, 59°55’W), Manaus, AM	**14**	**7II** 76.6 (111) **5II+IV** 23.4 (111)	**DAPI: ±T** 96.0 (302)		**T** 70.2 (37)	**+T** 13.0 (214)	

The chromosome preparations were obtained from the gonads of adult specimens, using the procedure described by Schneider et al. (2009a); the slides were stained with a 3% Giemsa solution. Constitutive heterochromatin was detected by C-banding (Sumner, 1972) and subsequently stained with DAPI (4`, 6-diamidino-2-phenylindole). Fluorescence *in situ* hybridization (FISH) was used to localize the 28S rDNA, (TTAGG)n telomeric sequence, and Cot-DNA fraction, following the technique of Pinkel et al. (1986), with the minor modifications described by Almeida et al. (2010).

Samples of muscle tissue from all the specimens were placed in microcentrifuge tubes and stored in a freezer at -80 ºC. The DNA was extracted using the commercial DNeasy Blood & Tissues kit (Qiagen). The 28S rDNA probes were obtained by PCR using the genomic DNA of *Brotheas amazonicus* and the primers 28S-F 5’ GACCCGTCTTGAAACACGG and 28S-R 5’ TCGGAAGGAACCAGCTACTT, described by Nunn et al. (1996). For telomeric-FISH, the primers Tel-F 5’ TAGGTTAGGTTAGGTTAGG and Tel-R 5’ AACCTAACCTAACCTAACC were used as probes, without a DNA template (Ijdo et al., 1991). The 28S rDNA and telomeric probes were labeled by PCR, using digoxigenin-16-dUTP (Roche) or biotin-11-dUTP (Roche), and detected, respectively, with anti-digoxigenin conjugated with rhodamine (Roche) and anti-biotin conjugated with Alexa-Fluor 288 (Thermo Fisher Scientific). The Cot-DNA fractions were obtained, following the protocol of Zwick et al. (1997), with the modifications suggested by Mello et al. (2014), using the genomic DNA of *B. amazonicus*, *Ischnotelson peruassu* and *Tityus metuendus*. This Cot-DNA was labeled with the DIG-Nick translation mix (Sigma-Aldrich) or Bio Nick DNA Labelling System (Thermo Fischer Scientific). All chromosome preparations were counterstained with VECTASHIELD antifade mounting medium with DAPI (Vector). The chromosome images were captured using a Zeiss Imager A2 microscope (100x objective lens and 2× Optovar magnification), coupled to a digital camera, equipped with the Axio Vision software. 

## Results

### Chromosome characterization

**Figure 1 -:**
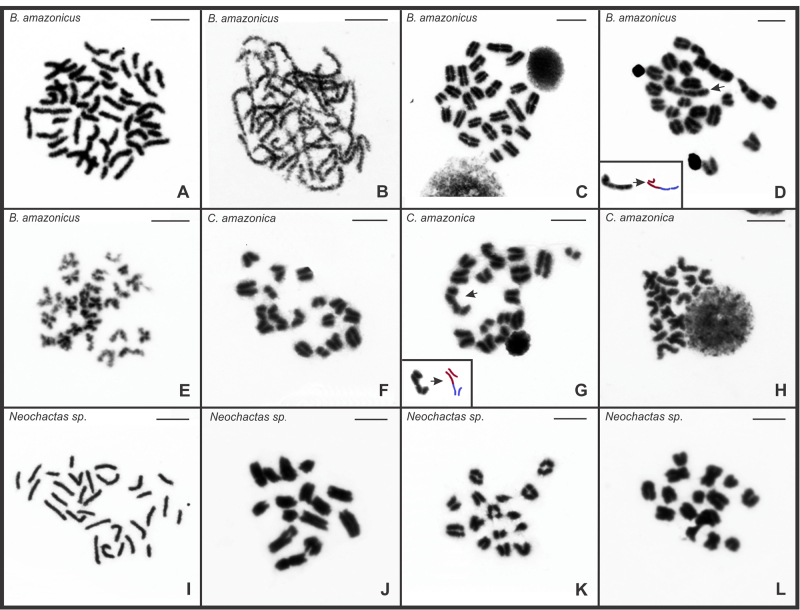
Testicular cells of Chactidae scorpions after Giemsa staining. (a-e) Cells of *B. amazonicus.* (a) Mitotic metaphase cells, with 2n=50. (b) Pachytene. (c-d) Postpachytene cells with 25II and 23II+IV, respectively. The insert in (d) is the schematic interpretation of the chain of four chromosomes. (e) Metaphase II with n=25. (g-h) Cells of *C. amazonica*. (f) Postpachytene cell with 18II. (g) Postpachytene cell with 16II+IV and schematic interpretation of the chain with four chromosomes. (h) Metaphase II cell with n=18. (i-l) Cells of *Neochactas* sp. (i) Mitotic metaphase with 2n=30. (j-k) Postpachytene cells, with 15II. (l) Metaphase II with n=15. II = bivalent. IV = chain of four chromosomes. Arrow = chromosome chain. Scale bar = 10 µm.

The chromosomes of all chactid species were identified as monocentric, given the presence of a primary constriction with a well-located centromere (Figure 1). The meiotic cells showed the achiasmatic behavior of the chromosomes. The mitotic metaphase cells of all specimens of *B. amazonicus* (Table 1) revealed 2n=50 (Figure 1A). The pachytene spermatocytes presented completely synapsed chromosomes (Figure 1B). In the specimens from the Adolpho Ducke Forest Reserve and one individual from Novo Airão, all postpachytene cells showed 25 bivalents (Figure 1C). But other males from Novo Airão presented 23 bivalents plus a chain of four chromosomes (23II+IV) in nine of the 75 postpachytene cells examined (Figure 1D). The metaphase II cells of all individuals exhibited n=25, with 15 meta/submetacentric and 10 acrocentric chromosomes (Figure 1E).

In *C. amazonica*, the diploid number 2n=36 was determined through the analysis of postpachytene and metaphase II cells. Most postpachytene nuclei presented 18 bivalents (Figure 1F), with the exception of 10 of the 75 cells analyzed, which had 16 bivalents plus a chromosomal chain of four elements, 16II+IV (Figure 1G). The metaphase II cells revealed n=18, including 13 acrocentric and five meta/submetacentric chromosomes (Figure 1H). *Neochactas* sp. presented 2n=30 (Figure 1I), including 10 pairs of meta/submetacentric chromosomes and five acrocentric (Figure 1I, L). The postpachytene and metaphase II cells showed 15 bivalents and n=15, respectively (Figure 1J, K, L).

**Figure 2 -:**
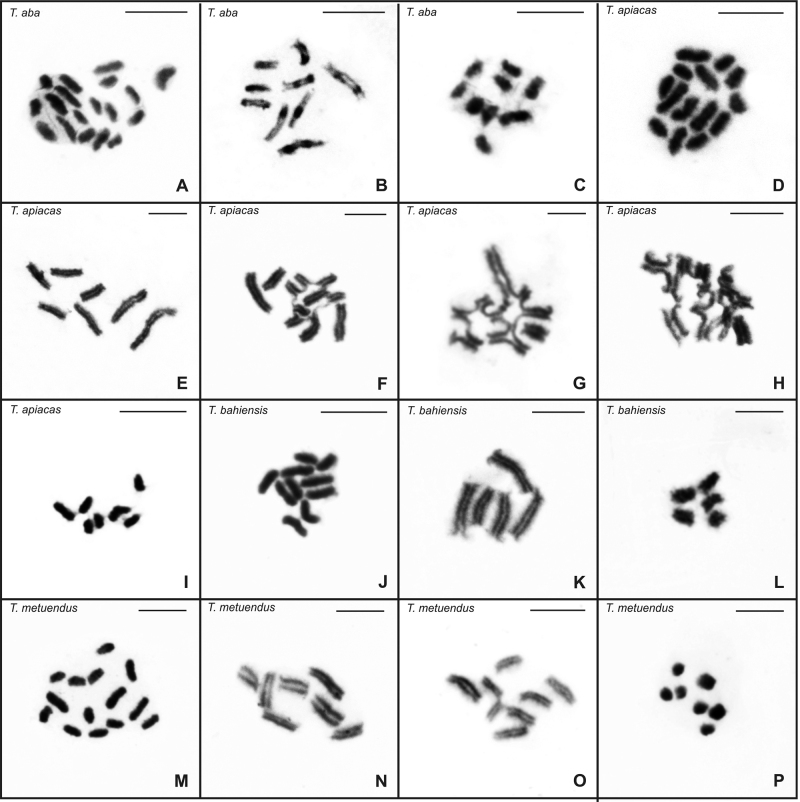
Testicular cells of Buthidae scorpions stained with Giemsa. (a-c) Cells of *T. aba.* (a) Mitotic metaphase, with 2n=18. (b) Postpachytene nuclei, with 9II. (c) Metaphase II cells, with n=9. (d-i) *Tityus apiacas* cells. (d) Mitotic metaphase, with 2n= 14. (e) Postpachytene nuclei, with 7II. (f-h) Postpachytene cells with high variability of multivalent chromosome associations. (i) Metaphase II cells, with n=7. (j-l) Cells of *T. bahiensis.* (j) Mitotic metaphase, with 2n=10. (k) Postachytene nuclei, with 5II. (l) Metaphase II cells, with n=5. (m-p) Cells of *T. metuendus*. (m) Mitotic metaphase, with 2n=14. (n-o) Postpachytene nuclei, with 7II and with 5II+IV respectively. (p) Metaphase II cells, with n=7. II = bivalent. IV = chain of four chromosomes.II = bivalent. IV = chain of four chromosomes. Scale bar = 10 µm.

The buthid scorpions exhibited holocentric chromosomes and absence of intraspecific variability in diploid number (Table 1). In some species, however, different chromosomal configurations were observed in postpachytene cells (Figure 2). *Tityus aba* showed 2n=18, with four large, four medium and 10 small-sized chromosomes (Figure 2A). Postpachytene nuclei exhibited nine bivalents and metaphase II cells n=9 (Figure 2B, C). *Tityus apiacas* presented 2n=14, including four large and 10 medium chromosomes that gradually decreased in size (Figure 2D). Some postpachytene cells revealed seven bivalents (Figure 2E), although approximately 80% of them presented a high variability of multivalent chromosome associations (Figure 2F, G, H). In these cells, the number of chromosomes of the chains was not determined due to the complexity of the configurations. The metaphase II cells always showed n=7 (Figure 2I).

Mitotic metaphase cells of *T. bahiensis* exhibited 2n=10, with eight medium and two small-sized chromosomes (Figure 2J). Postpachytene nuclei invariably presented five bivalents (Figure 2K) and metaphase II cells n=5 (Figure 2I). In all individuals of *T. metuendus*, the mitotic metaphase cells exhibited 2n=14, including four large and 10 medium/small-sized chromosomes (Figure 2M). In specimens from Adolpho Ducke Forest Reserve and two males from UFAM, pachytene and postpachytene cells showed seven bivalents (Figure 2N). However, in the other males from UFAM, the postpachytene nuclei revealed five bivalents plus a chain of four chromosomes (5II+IV) (Figure 2O). Metaphase II cells presented n=7 in both studied populations (Figure 2P).

### Chromosome banding and *in situ* hybridization

**Figure 3 -:**
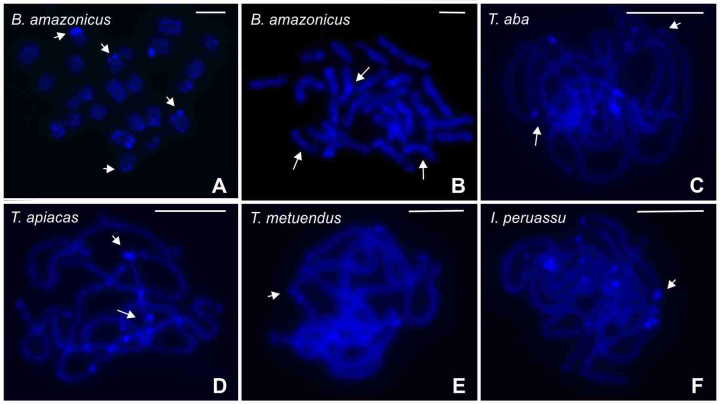
Chromosomes of Chactidae and Buthidae scorpions stained with DAPI C-banding (a) and DAPI after FISH (b-f). (a-b) Postpachytene cells, showing terminal (small arrow) and interstitial (large arrow) heterochromatin, respectively. (c-f) Pachytene nuclei with terminal and interstitial heterochromatic regions. Scale bar = 10 µm.

Cytogenetic preparations of all chactid species were submitted to C-banding plus DAPI, but only *B. amazonicus* produced positive signals. In this species, blocks of constitutive heterochromatin were observed in one or both chromosome ends of at least 10 bivalents (Figure 3A). However, the morphology of the heterochromatin-bearing chromosomes was not identified because the analyses were based mainly on postpachytene cells.

**Figure 4 -:**
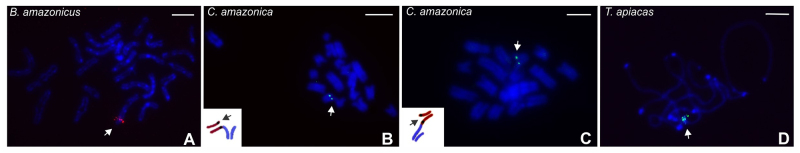
Localization of 28S rDNA gene in Chactidae and Buthidae scorpions. (a) Postpachytene, showing rDNA genes in the subterminal region of one bivalent. (b-c) Postpachytene cells, revealing rDNA sites in chromosome of the chainand schematic representation of the multivalent, showing the localization of the 28S rDNA sites. (d) Pachytene with terminal rDNA genes. Scale bar = 10 µm.

The distribution of DAPI bands observed after the FISH was variable in the species herein analyzed (Figure 3B, C, D, E, F). *Brotheas amazonicus* revealed, in addition to the terminal heterochromatin, positive signals in the interstitial regions of some chromosomes (Figure 3B). In the *Tityus* species, tenuous signals were visualized in the terminal and interstitial regions of the chromosomes of *T. aba* and *T. apiacas* (Figure 3C, D), and only in the terminal regions of the chromosomes of *T. metuendus* and *I. peruassu* (Figure 3E, F). In *T. bahiensis*, no evidence of heterochromatin was observed in cells stained with DAPI after FISH (not shown). FISH with the 28S rDNA probe revealed two chromosomes with ribosomal cistrons in *B. amazonicus* and *C. amazonica* (Figure 4A, B, C). However, in *B. amazonicus*, the rDNA sites were located in the subterminal region of one bivalent (Figure 4A) while in *C. amazonica*, these sites occurred in the terminal region of two chromosomes of the chain (Figure 4B, C). In the *Tityus* species, the 28S rDNA genes were only identified in *T. apiacas*, which presented bright signals in the terminal region of one bivalent (Figure 4D).

**Figure 5 -:**
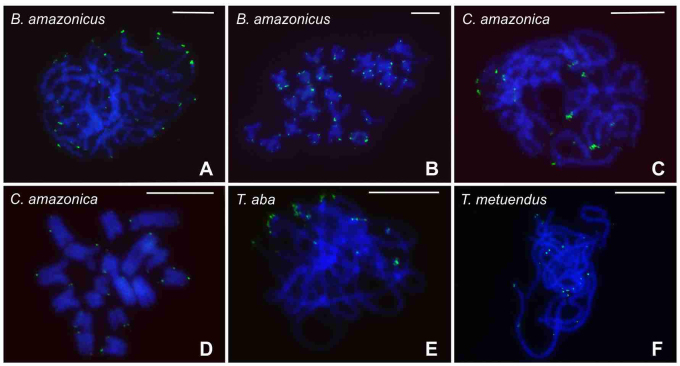
Localization of (TTAGG)ntelomeric sequence in Chactidae and Buthidae scorpions. (a-b) Pachytene and metaphase II cells, respectively. (c-d) Pachytene and postpachytene cells, respectively. (e-f) Pachytene nuclei. Scale bar = 10 µm.

Mitotic and meiotic cells of *B. amazonicus, C. amazonica, T. aba* and *T. metuendus* were analyzed using FISH with the (TTAGG)n probe, which revealed typical telomeric signals in the terminal regions of the chromosomes (Figure 5). No evidence of positive labeled sites was observed in the chromosome interstitial regions of these investigated species.

**Figure 6 -:**
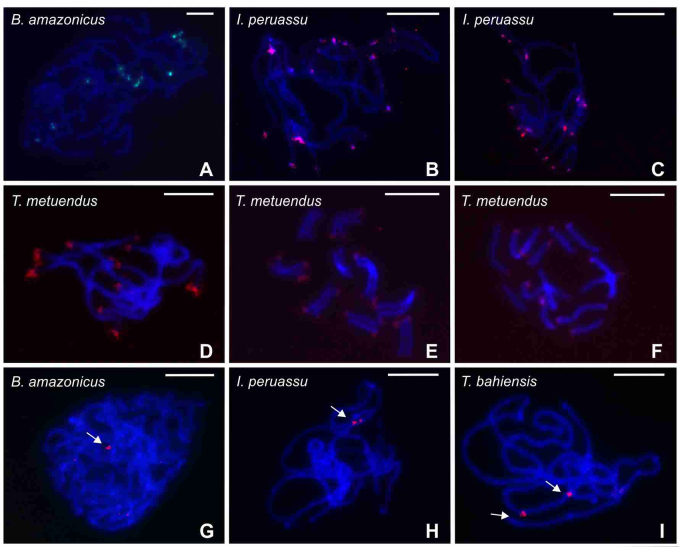
Localization of Cot-DNA fraction in Chactidae and Buthidae scorpions. (a-f) Hybridization with species-specific probes. (g-i) Hybridization with probes of Cot-DNA fraction of *T. metuendus*. (a-f) Observe the signals in the terminal region of the chromosomes. (g-i) Note tenuous signals (arrows) in the interstitial region of the chromosomes. Scale bar = 10 µm.

The Cot-DNA obtained from *B. amazonicus*, *I. peruassu* and *T. metuendus* revealed species-specific signals in these scorpions (Figure 6). However, only in *T. metuendus*, the labeled regions were strong and well-defined, being located in the terminal regions of all chromosomes (Figure 6D, E, F). The Cot-DNA fraction of *T. metuendus* was used as probe in the chromosome preparations of *B. amazonicus*, *I. peruassu*, *T. aba*, *T. apiacas* and *T. bahiensis*, showing tenuous signals in the interstitial regions of the chromosomes (Figure 6G, H, I); except in *T. aba* and *T apiacas*, in which positive signals were not observed.

## Discussion

The cytogenetic analyses presented herein expanded the available data for the family Chactidae from one to three genera and revealed, for the first time, the presence of monocentric chromosomes and achiasmate meiosis. The diploid number 2n=50 observed in *B. amazonicus* is the same previously recorded by Ferreira (1968). The 2n=36 of *C. amazonica* and 2n=30 of *Neochactas* sp*.* are the lowest diploid numbers already described for Chactidae and closely-related families (*sensu* Santibáñez-López et al., 2019), such as the Scorpiopidae (2n=48-147) and Euscorpiidae (46-112) (Schneider et al., 2023). 

Buthidae is the family with the most distinct chromosome characteristics among the 10 others cytogenetically investigated up to now, given the presence of holocentric chromosomes and the lowest diploid numbers, 2n=5-56 (Schneider et al., 2023). The 2n=10 herein established for all individuals of *T. bahiensis* is the third most frequently recorded for this species, that have been identified in more than five Brazilian populations (Schneider *et al.*, 2023). This species exhibited an intriguing variability in diploid number, with 13 distinct karyotype formulae (2n=5, 6, 7, 9, 10, 12, 13, 14, 15, 17, 18, 19 and 20) already recorded (Schneider *et al.*, 2023). Piza (1940) initially proposed that this diversity of diploid number in *T. bahiensis* could be related to interpopulational variations. However, Adilardi et al. (2020) suggested 2n=18 as the ancestral diploid number for this species. The 2n=10 is a chromosome number observed in geographically intermediate populations from Brazil and it would have supposedly originated due to fusion events, with Northern populations presenting higher diploid number (2n=17-20) than Southern populations (2n=12-15). However, it seems that independent events of hybridization could have originated the 2n=10 in the specimens of *T. bahiensis*, considering the different chromosome configurations observed during meiosis, i.e., only bivalents, such as the ones registered here, or chromosomal chains composed of three, four, six, eight or 10 chromosomes. 

*Tityus aba* has a relatively large diploid number (2n=18). Within the species of the subgenus *Tityus*, this diploid number was only described in some populations of *T. bahiensis* (Piza, 1949). Based on the color pattern and geographic distribution, some species of the subgenus *Tityus* have been grouped into complexes. One of these complexes is *T. stigmurus* (Souza et al., 2009), which includes *T. aba* and three other cytogenetically analyzed species, *Tityus martinpaechi* with 2n=6, *Tityus serrulatus* with 2n=12, and *Tityus stigmurus* with 2n=14 (Piza, 1950; Schneider and Cella, 2010; Mattos et al., 2013; Lima et al., 2020). If the *T. stigmurus* complex really corresponds to a monophyletic group, the diploid number is extremely variable among these closely-related species. However, a phylogenetic analysis is still necessary to test the validity of this group of species.

*Tityus apiacas* and *T. metuendus* exhibited the same diploid number, 2n=14; but this latter species differed from the 2n=15-16 previously recorded by Piza (1952). Both species belong to the subgenus *Atreus*, composed mainly of dark-colored large-sized Amazon species (Lourenço, 2006). Only four other *Atreus* species have been cytogenetically analyzed, *Tityus fuhrmanni* (2n=22), *Tityus magnimanus* (2n=20), *Tityus obscurus* (2n=11-16), and *Tityus ythieri* (2n=20) (Kovařík et al., 2009; Almeida et al., 2017). In a molecular study that included some *Tityus* species of the subgenera *Archaeotityus*, *Atreus*, and *Tityus*, monophyly only of the subgenus *Tityus* was not recovered (Ojanguren-Affilastro et al., 2017a). Considering the diversity of species included in this subgenus and the scarcity of cytogenetic studies, any discussion about the chromosome evolution of this group is premature. 

The chromosome chain observed during the meiosis in *B. amazonicus* and *C. amazonica* could have been the result of reciprocal translocation, involving small fragments of the chromosome ends of two non-homologous elements. Alternatively, taking into account the absence of chromosome chain in all the cells of a given individual and the maintenance of the diploid number and chromosome morphology, this configuration may reflect an association between non-homologous chromosomal regions. On the other hand, in *T. metuendus*, the chromosome chain was observed in all cells of the two individuals and it has probably originated as a result of heterozygous translocation, involving regions of non-homologous chromosomes. This rearrangement resulted in the formation of a quadrivalent association during the meiosis I, but the diploid number has not changed. In *T. apiacas*, a variable degree of synapses should be responsible for the presence of bivalents and chromosome chains with a variable number of chromosomes among the cells of the same individual. Similar scenarios have been reported in other scorpions, such as *Ischnotelson guanambiensis*, *Jaguajir pintoi*, *T. bahiensis*, *Tityus paraguayensis* and *Tityus pusillus* (for revision see Schneider et al., 2009b; Mattos et al., 2013, 2018; Ubinski et al., 2018). 

The lack of positive C-band regions in *C. amazonica* and *Neochactas* sp. indicates that the chromosomes of these species contain a smaller quantity of constitutive heterochromatin when compared to *B. amazonicus* that revealed positive DAPI C-bands in the terminal regions of various chromosomes. In scorpions, C-banding plus DAPI reveals better contrasted bands when compared to C-banding plus Giemsa (Mattos et al., 2013; Adilardi et al. 2020). However, the data obtained in *B. amazonicus* cannot be compared to the C-banding pattern described for other scorpions with monocentric chromosomes (Shanahan, 1989; Schneider et al., 2009a), considering that the analysis of quantity and distribution of heterochromatin were accomplished with distinct chromosome staining. Moreover, the base composition of the C-banded region of *B. amazonicus* is not necessarily AT-rich, as pointed by Barros *et al.* (2010) in a study using different staining methodologies after C-banding. 

Similar to the observations of this work, the presence of (TTAGG)n telomeric sequence in scorpions has been found in species with monocentric or holocentric chromosomes and only in the terminal regions, even in rearranged chromosomes (Adilardi et al., 2015, 2016; Almeida et al., 2017; Ojanguren-Affilastro et al., 2017b; Mattos et al., 2018; Šťáhlavský et al., 2018; Ubinski et al., 2018). Despite the scorpions investigated here have shown very different karyotypes, in species of both Chactidae and Buthidae families, the rDNA sites were located in the terminal regions of two chromosomes. These findings diverge from the pattern observed in other groups of animals and plants, in which species with similar karyotype characteristics often present variation in the number or location of the rDNA sites (Datson and Murray, 2006; Bomborová et al., 2007; Cabrero and Camacho, 2008; Catroli et al., 2011). 

The Cot-DNA fraction has been useful for studies of evolution and karyotypic diversity, given that the repetitive DNA sequences play an important role in the modification of the genome (Elder and Turner, 1995). Additionally, structural chromosome rearrangements could be associated with regions of constitutive heterochromatin, which are composed of different types of repeated sequences (Badaeva et al., 2007). The data obtained in the present study indicate that these scorpion species have a small quantity of this class of repetitive DNA, considering that the FISH-Cot signals were small and not distributed along the chromosome length. The exception was *T. metuendus*, in which the Cot-DNA fraction hybridized with the terminal regions of all chromosomes. This pattern was similar to the one previously described for *T. obscurus*, that evidenced conspicuous labeled regions in all chromosome ends (Almeida et al., 2017). In some cases, however, the scarcity of the moderate or high repeated sequences detected by the Cot-DNA fraction does not reflect the absence of other types of repetitive sequence, such as the microsatellites. The identification and localization of microsatellite sequences has been employed in the analysis of some species of insects (Kubat et al., 2008; Cuadrado and Jouve, 2010; Mello et al., 2014; Milani and Cabral-de-Mello, 2014; Gusso-Goll et al., 2015) and has been useful for distinguishing populations (Micolino et al., 2019). The use of Cot-DNA fraction of *T. metuendus* against the chromosome preparations of other chactids and buthids revealed that the repetitive sequences are not shared among the species. Rapid modifications of the repetitive DNA may generate species-specific sequences, resulting in variations, even between closely-related organisms (Miklos, 1985). 

In the species investigated in this work, the conserved localization of rDNA genes and telomere sequences, contrasted with the results obtained by C-banding, DAPI after FISH, and Cot-DNA fraction, which indicated a variable pattern of the distribution of these regions, that is, (i) positive heterochromatin and Cot-DNA signals (*B. amazonicus* and *I. peruassu*), (ii) small blocks of heterochromatin with large Cot-DNA signals (*T. metuendus*), (iii) positive heterochromatic regions and absence of Cot-DNA signals (*T. aba* and *T. apiacas*), and (iv) negative heterochromatin and Cot-DNA signals (*T. bahiensis*). A comparative analysis among species with monocentric and holocentric chromosomes and with or without rearranged chromosomes still did not reveal a clear relation with the quantity of heterochromatin. All these findings indicate that the repetitive regions in scorpion chromosomes are heterogeneous and must be analyzed using different cytogenetic approaches. In addition, more systematic data on the quantity and types of repetitive sequences found in the scorpion genome will be necessary to determine whether a relationship exists between the occurrence of these regions and the rates of chromosomal rearrangement found in the different species. *Tityus bahiensis*, however, is still the scorpion with the highest variation of chromosome number already registered and the lowest quantity of repetitive regions using distinct techniques.
